# The impact of body armor on physical performance of law enforcement personnel: a systematic review

**DOI:** 10.1186/s40557-017-0169-9

**Published:** 2017-05-16

**Authors:** Colin Tomes, Robin Marc Orr, Rodney Pope

**Affiliations:** 1 Department of Health, Nutrition and Exercise Science, Messiah College, Grantham, PA USA; 2 Tactical Research Unit, Bond University, Gold Coast, QLD 4226 Australia

**Keywords:** Officer, Police, Body armour, Personal protective equipment

## Abstract

**Background:**

The law enforcement officer profession requires performance of arduous occupational tasks while carrying an external load, consisting of, at minimum, a chest rig, a communication system, weaponry, handcuffs, personal protective equipment and a torch. The aim of this systematic review of the literature was to identify and critically appraise the methodological quality of published studies that have investigated the impacts of body armour on task performance and to synthesize and report key findings from these studies to inform law enforcement organizations.

**Methods:**

Several literature databases (Medline, CINAHL, SPORTDiscus, EMBAS) were searched using key search words and terms to identify appropriate studies. Studies meeting the inclusion criteria were critically evaluated using the Downs and Black protocol with inter-rater agreement determined by Cohen’s Kappa.

**Results:**

Sixteen articles were retained for evaluation with a mean Downs and Black score of 73.2 ± 6.8% (k = 0.841). Based on the research quality and findings across the included studies, this review determined that while effects of body armour on marksmanship and physiological responses have not yet been adequately ascertained, body armour does have significant physical performance and biomechanical impacts on the wearer, including: a) increased ratings of perceived exertion and increased time to complete functional tasks, b) decreased work capability (indicated by deterioration in fitness test scores), c) decreased balance and stability, and d) increased ground reaction forces.

**Conclusions:**

Given the physical performance and biomechanical impacts on the wearer, body armour should be carefully selected, with consideration of the physical fitness of the wearers and the degree to which the armour systems can be ergonomically optimized for the specific population in question.

## Background

While a large percentage of a Law Enforcement Officer’s (LEO) time on duty is spent in sedentary activities, a high level of physical fitness remains necessary to effectively respond to many incidents [1]. Although incident responses are sporadic, a LEO will inevitably be required to run, jump, crawl, balance while moving quickly, engage in combat, climb, lift, and push or pull significant weights in the execution of their duties, often without warning [1, 2]. Additionally, the LEO profession requires performance of these arduous occupational tasks while carrying an external load, consisting of, at minimum, a chest rig, a communication system, weaponry, handcuffs, personal protective equipment (PPE) and a torch [3].

With an increasing focus on minimizing the risks associated with the performance of these demanding tasks in unpredictable and potentially hostile environments, the weight of these external loads carried by LEO has been steadily increasing [4]. Total weight of external loads can range from approximately 3.5 kg [3] for the general duty officer to 22 kg in specialist police [5] and may even exceed 40 kg in certain circumstances [6]. This upward trend in carried loads is due not only to the increasing complexity of modern tactical engagements [7], but also to the increased emphasis on improving survival without reducing unit mobility [8]. Given that additional load in the form of body armour is known to be effective in reducing fatalities in military environments, use of body armour is becoming more widespread amongst LEO. For example, one Police Force in New Zealand is citing the escalation of violent crimes as evidence of the need to regularly equip LEOs with body armour, particularly for protection against stab threats [3].

While increasing numbers of law enforcement organizations have been making the decision to issue body armour systems, the additional external load associated with this equipment has been associated with increased rates of injury and reduced operational capabilities in tactical populations [9, 10]. In research, primarily on military personnel, load carriage has been found to cause musculoskeletal injuries (e.g. back pain, lower limb stress fractures), neurological injuries (e.g. brachial plexus palsy) and integumentary injuries (e.g. chaffing, blisters) [11]. Chronic increases in levels of physical exertion due to additional occupational load carriage may even be a causative factor in illness, as a result of depressed immune function [12], especially in LEO with a lower body mass or lower level of physical fitness [13]. Impairments to mission capability and performance can also result from load carriage, due to reduced carrier mobility [14] and an increase in time required to complete functional tasks [3, 15]. These impairments can impact on aspects of marksmanship [5, 16–20] and attention-to-task [21, 22] and increase the physiological cost to complete a task [23] when on duty. Essentially, while body armour provides protection to the wearer, it will, like other types of loads, also impart both risks and physiological costs.

With the majority of body armour research having been conducted in military populations, and despite the increasing use of body armour systems by LEO, published research that has examined the extent to which body armour can impact the performance capabilities of LEO during physically demanding occupational tasks has not been comprehensively and critically reviewed. The aim of this systematic review of the literature was therefore to identify and critically appraise the methodological quality of published studies that have investigated the impacts of body armour on task performance in tactical populations, and to synthesize and report key findings from these studies to inform law enforcement organizations.

## Methods

In order to identify and obtain relevant original research for review, key literature databases were systematically searched using specific keywords of relevance to the topic. The selection of keywords to be used in the systematic search was guided by review of keywords used in a sample of relevant articles. The databases searched, specific key words, and search strategies employed are detailed in Table 1. To improve the relevance of search results, filters that reflected study eligibility criteria were applied in each database, where available. In databases where these filters were unavailable or were only partially available, the study eligibility criteria were applied manually through screening of study titles and abstracts. The eligibility criteria were subsequently applied to the full-text of identified articles that were not excluded during the screening of titles and abstracts, to select a final set of eligible articles for inclusion in the literature review. The results of the search, screening and selection processes were documented in a PRISMA flow diagram [24].

**Table 1:** Keywords, subject headings and arrangement of terms searched by database

Database	Terms				
	body armour		Population		Outcome measures
Medline Complete	(armour OR armor OR “ballistic vest” OR “tactical vest” OR “bulletproof vest” OR “ballistic vests” OR “tactical vests” OR “bulletproof vests” OR “protective clothing”)	AND	(“Police”[Mesh] OR “Military Personnel”[Mesh] OR “Military Science”[Mesh] OR “Military Medicine”[Mesh] OR military OR soldier OR officer OR police OR law enforcement OR Army OR Navy OR naval OR “Armed Forces” OR security OR Guard* OR tactic* OR combat OR “special forces” OR “special operations” or defense OR defence)	AND	(“Exercise”[Mesh] OR “Physical Exertion”[Mesh] OR “Physical Fitness”[Mesh] OR “Work Performance”[Mesh] OR “Task Performance and Analysis”[Mesh] OR “Biomechanical Phenomena”[Mesh] OR exercise OR exertion OR performance OR biomechanic* OR endurance OR fitness OR strength OR conditioning OR “movement analysis” OR kinematic* OR mechanic* OR task* OR training)
CINAHL	(armour OR armor OR “ballistic vest” OR “tactical vest” OR “bulletproof vest” “ballistic vests” OR “tactical vests” OR “bulletproof vests” OR “protective clothing”)	AND	((MH “Police”) OR (MH “Military Personnel”) OR (MH “Military Recruits”) OR (MH “Research, Military”) OR (MH “Military Training”) OR (MH “Military Services”) OR (MH “Military Medicine”) OR military OR soldier OR officer OR police OR law enforcement OR Army OR Navy OR “Armed Forces” OR security OR Guard* OR tactic* OR combat OR “special forces” OR “special operations” or defense OR defence))	AND	((MH “Exercise”) OR (MH “Resistance Training”) OR (MH “Physical Fitness”) OR performance OR tactic* OR biomechanic* OR exertion OR endurance OR fitness OR strength OR conditioning OR “movement analysis” OR kinematic* OR mechanic* OR task* OR training)
SPORTDiscus	(armour OR armor OR “ballistic vest” OR “tactical vest” OR “bulletproof vest” OR “ballistic vests” OR “tactical vests” OR “bulletproof vests” OR “protective clothing”)	AND	(military OR soldier OR officer OR police OR law enforcement OR Army OR Navy OR naval OR “Armed Forces” OR security OR Guard* OR tactic* OR combat OR ”special forces” OR “special operations” or defense OR defence)	AND	(exercise OR exertion OR performance OR biomechanic* OR endurance OR fitness OR strength OR conditioning OR “movement analysis” OR kinematic* OR mechanic* OR task* OR training)
EMBASE	(‘body armor’/exp OR armour OR armor OR “ballistic vest” OR “tactical vest” OR “bullet proof vest” OR “ballistic vest” OR “tactical vest” OR “bulletproof vest” OR “protective clothing”)	AND	(‘military phenomena’/exp OR ‘police’/exp OR military OR soldier OR officer OR police OR law enforcement OR Army OR Navy OR naval OR “Armed Forces” OR security OR Guard* OR tactic* OR combat OR “special forces” OR “special operations” or defense OR defence)	AND	(‘exercise’/exp OR ‘physical performance’/exp OR ‘athletic performance’/exp OR ‘biomechanics’/exp OR ‘kinematics’/exp OR ‘training’/exp OR exercise OR exertion OR performance OR biomechanic* OR endurance OR fitness OR strength OR conditioning OR “movement analysis” OR kinematic* OR mechanic* OR task* OR training)

The specific inclusion criteria applied for this review were: a) human subjects, b) English language availability, c) peer reviewed publications reporting original research for the first time; d) publication date between 1 January 1991 and 17 June 2016; and e) investigated effects of body armour on at least one of the following outcomes of direct relevance to LEO: occupationally-relevant physical performance; levels of task-related physical exertion; mobility; balance; biomechanics; cognitive performance; or marksmanship. Subject matter experts were consulted in determining the date range. Studies published prior to 1991 were excluded because they preceded the Gulf War (having commenced in 1991) which substantially changed individual combat systems (increased use of body armour) and changed operating conditions from those of previous conflicts. Identified publications that fell outside the date range of the review were excluded from the critical review but used to provide context, where useful. The exclusion criteria employed in assessing eligibility of individual studies for inclusion in the literature review were: a) studies that did not measure at least one aspect of human performance; b) studies that involved testing personal protective equipment (PPE) used in Chemical, Biological, Radiological, Nuclear, and high-yield Explosive (CBRNE) environments, concealable or soft body armour only, or a complete load carriage or fighting load; c) studies in extreme environments (high altitude, very cold, etc); and d) studies in which use of body armour was not a primary independent variable.

Eligible publications identified through the literature search, screening and selection processes were then critically appraised to assess methodological quality using the Downs and Black scoring system [25]. This approach to critical appraisal was chosen for the following reasons: a) it was initially developed using psychometrics, b) repeated testing has demonstrated good reliability and validity of this approach, and c) its results are amenable to translation into a methodological quality rating such as the Kennelly grading system used in this review [26], as detailed below. To assess each article with the Downs and Black protocol, 25 of 27 questions, covering statistical methods, validity, bias, data reporting, power analysis, and organization were scored as either ‘0’ points (if the methodological details requested in the question were absent or undeterminable, or ‘1’ point (if the methodological details were reported correctly). Two further questions, Question 5 (relating to potential confounding variables) and Question 27 (relating to sample size calculations and statistical power analysis), are typically scored across a range. Question 5 is scored on a scale from ‘0’ points to ‘2’ points (based on the quality of reporting of confounding factors) while Question 27 is scored on a scale from ‘0’ points to ‘5’ points (based on the extent to which statistical power analyses have informed sample size decisions). While the ‘0’ to ‘2’ scale was retained in this review for Question 5, the review utilized a modification to Question 27 of the Downs and Black protocol first proposed by Eng, et al. [27]. Eng, et al. [27] suggest that Question 27 is ambiguously worded, and so recommend scoring this question regarding statistical power calculations dichotomously, as a correction. This modification ensures the impact this question can have on a publication’s final total raw score is reduced, limiting the impact of subjectivity in the assessment. For comparison purposes and to improve reporting, the critical appraisal score (CAS) for each included study was converted to a percentage score and reported to one decimal place. The CAS was calculated by dividing the actual raw modified Downs and Black score by the highest possible raw score (modified to 28 points) and then multiplying this figure by 100 to convert this proportion to a percentage.

To obtain a quality grade for each research paper, the CAS was then compared to the grading system for methodological quality proposed by Kennelly [26]. Kennelly’s grades are categorized as ‘poor’, ‘fair’ and ‘good’, with the methodological quality rating determined by the scores assigned in the modified Downs and Black protocol. The grade categories and associated ranges of raw Downs and Black scores employed by Kennelly are: 14 or fewer points, classified as ‘poor’ quality research, 15–19 points, classified as ‘fair’ quality research, and 20 or more points, classified as ‘good’ quality research. However, as the Kennelly system was based on the original Downs and Black protocol (which had a maximum possible score of 32 points), in this review (in which the total possible score from the modified Downs and Black protocol was 28 points) the Downs and Black scores associated with each of Kennelly’s grades were converted to percentages. On this basis, the final grading system employed in this review for grading methodological quality of each of the included studies was as follows: Downs and Black score <45.4% - study classified as being of ‘poor’ methodological quality, score of 45.4–61.0% - study classified as being of ‘fair’ methodological quality, and score >61.0% - study classified as being of ‘good’ methodological quality.

All studies were independently critically appraised using the Downs and Black protocol by two authors (CT, RO), with the level of agreement between the appraisers measured using a Cohen’s Kappa (k) analysis of all raw scores (27 scores per paper). For final scores, any disagreements between the two authors (CT, RO) in scores awarded were settled by consensus or, if necessary, adjudication by the third author (RP).

Following the critical appraisal process, key data were extracted from the included papers and compiled in tables to provide a concise and systematic overview of key attributes of, and findings from, included studies. The key table headings were a) title, lead author and publication date, b) demographics of the participants, c) the equipment used and worn, d) key independent and dependent variables, e) the occupational task or test employed to assess performance and f) the CAS, expressed as a percentage. Key outcomes were also extracted and compiled, although only those outcomes which reached statistical significance were included, for brevity, in the table, as indicators of significant impacts of the body armour on the occupationally-relevant task performance of the wearer. Study findings from across the included studies were then critically synthesized to generate a clear summary of key findings and identify the strength of evidence supporting these key findings.

## Results

**Fig. 1:**
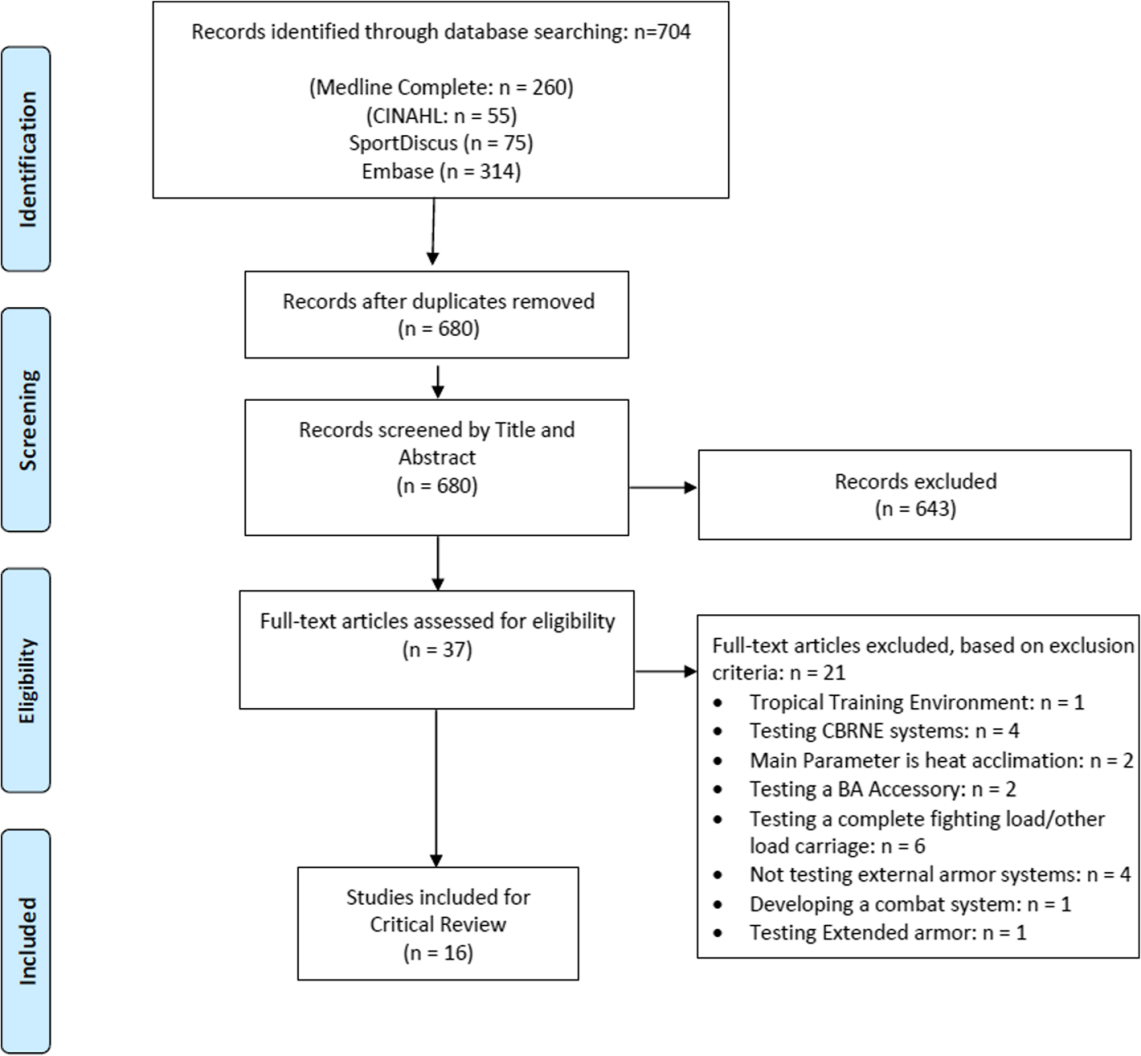
PRISMA diagram detailing search process

The PRISMA diagram (Fig. 1) details the results of the literature search, screening, and selection processes. In total, the initial search yielded 704 publications, from which 24 duplicates were removed. Of the remaining 680 publications, 37 were considered to be of potential relevance following initial screening and were examined in full text to further assess eligibility. Ultimately, 16 eligible studies were retained and formed the basis for this review.

The research reported in this review originated from six countries. Specifically, six studies were from the US [23, 28–32], five publications were from Australia [5, 14, 33–35], two from New Zealand [3, 36], one from India [37], one from Poland [38], and one from the UK [39]. Three distinct subject populations were represented, consisting of university students (*n* = 7) [29, 31–35, 39], LEO (*n* = 4) [3, 5, 14, 36], and military personnel (*n* = 5) [23, 28, 30, 37, 38]. Additionally, ten of the publications studied only males [3, 5, 14, 33–39] and six included both males and females [23, 28–32]. The mean ± SD age across all studies was determined to be 28.85 ± 2.32 years, ranging from 21.9 ± 2.4 years [29] to 37 ± 9.16 years [3, 36].

The mean ± SD CAS, derived from critical appraisal of the methodological quality of each included study, was 73.2 ± 6.8%, ranging from 60.7% [35] to 85.7% [36]. This mean indicates that most studies were of at least ‘good’ (62.5%) methodological quality. The kappa statistic for inter-tester agreement of the methodological quality of the studies indicated an ‘almost perfect’ agreement (k = 0.841) [40]. When applying the Downs and Black protocol for critical appraisal of each publication, Questions 1–14, concerning how data was reported, generally scored highly across all studies. Low external validity scores (Questions 11–13) were attributed to those studies which did not recruit participants from a tactical population [29, 31–35, 39]. Additionally, one study recruited subjects from a population of military personnel (US Airborne Infantry Soldiers) that had been trained at a level substantially beyond their peers (US Infantry Soldiers). Questions 15–19, relating to internal validity of the study results, showed overall poor scores with respect to blinding, because in most cases, the test condition was a loaded state and was compared to an unloaded control state. The fact that these conditions did not allow for blinding of either the participants or the researchers who conducted and recorded measurements, accounts for the low scores in this area. However, those studies using within-subject measures were able to eliminate selection bias. Scores relating to whether there was adequate consideration of statistical power were generally low across the included studies, due either to insufficient reporting of power or sample size analyses, or low participant numbers.

The key data extracted from the included study reports are summarized in Table 2. All but one included study compared outcomes from a loaded condition with those from an unloaded control condition [38]. The anomalous study, by Majchrzycka et. al.[38], used the current Polish military armour plate as a control for comparison with new alternative plates. Additional independent variables were also incorporated in some of the included studies, including a fatigued test subject state [3, 36, 39] and the comparison of different armour configurations [35, 38]. Seven unique categories were identified among included studies based on the outcomes they assessed. These categories were: Physiological measures (*n* = 8) [23, 28, 29, 33, 34, 36, 37, 39], marksmanship measures (*n* = 1) [5], mobility measures (*n* = 2) [3, 14], kinetic and kinematic measures (*n* = 6) [3, 31, 32, 36], cognitive measures (*n* = 3) [33, 38, 39], subjective measures (*n* = 4) [28, 33, 34, 36], and thermal measures (*n* = 2) [33, 34]. Because some studies reported multiple outcome measures, they fitted into multiple categories and thus are referenced more than once in the categorized results presented on the page following.

**Table 2:** Summary of critically reviewed literature by author and title

Title/Author	Participants	Equipment	Variables	Intervention	Main Findings	CAS ^a^ (%)
Caldwell, et. al. 2011 [33] The interaction of body armour, low-intensity exercise, and hot-humid conditions on physiological strain and cognitive function	*n* = 9 Mean ± SD Age: 27.3 ± 5.43 years Mean ± SD Height: 180.3 ± 5.11 cm Mean ± SD Weight: 79.4 ± 11.3 kg Healthy, Active Male University students (AU ^b^)	AU standard issue kevlar vest with ceramic plate inserts (6.07 kg) Rabintex industries ballistic helmet (1.29 kg)	IND ^d^: Loaded or unloaded condition, environmental conditions (Hot-Humid) DEP ^e^: Body temp, HR ^k^, RPE^f^, vigilance, reaction time	2.5 h simulated patrol in hot-humid conditions	• Loaded condition did not induce heat illness (no core temp. >39.5 °C) • Progressive divergence between loaded and unloaded cardiovascular strain (*p* = 0.01) • Armour significantly increases RPE ^f^ (*p* = 0.02) • No significant change in cognitive function between loaded &unloaded conditions (*p* > 0.05)	67.9
Carbone, et. al. 2014 [5] The impact of load carriage on the marksmanship of the tactical police officer: a pilot study	*n* = 6 Mean ± SD Age: 33.3 ± 4.13 years Mean ± SD Height: 177.0 ± 11.8 cm Mean ± SD Weight: 89.2 ± 13.2 kg Mean ± SD years of experience in the police force: 10.9 ± 5.1 year Active male officers of a police Tactical Operations Unit (AU ^b^)	Body armour, helmet, primary (M4) and secondary (9 mm Glock) weapons (22.8 ± 1.8 kg)	IND ^d^: Loaded or unloaded condition DEP ^e^: Marksmanship accuracy	Police marksmanship test: one static shoot, and one tactical mobile shoot	• No significant difference in shooting results between loaded and unloaded conditions (*p* > 0.05)	71.4
Carlton, et. al. 2014 [14] The Impact of Occupational Load Carriage on the Mobility of the Tactical Police Officer	*n* = 6 Mean ± SD Age: 33.3 ± 4.13 years Mean ± SD Height: 177.0 ± 11.8 cm Mean ± SD Weight: 89.2 ± 13.2 kg Mean ± SD years of experience in the police force: 10.9 ± 5.1 year Active male officers of a police Tactical Operations Unit (AU ^b^)	Body armour, helmet, primary (M4) and secondary (9 mm Glock) weapons (22.8 ± 1.8 kg)	IND ^d^: Loaded or unloaded condition DEP ^e^: Speed and mobility during test course	10 m in line sprint 25 m simulated patrol Two 10 m dummy (70 kg) drags	• Time to complete the tactical movement task increased with load (mean ± SD time unloaded = 18.59 ± 2.44 s: loaded = 19.89 ± 1.61 s) and officers were significantly slower (*p* < 0.01) during the dummy drag mobility task when loaded (mean ± SD time unloaded = 9.29 ± 0.53 s: loaded = 10.25 ± 0.77 s)	71.4
Dempsey, et. al. 2013 [3] Impact of police body armour and equipment on mobility	*n* = 52 Mean ± SD Age: 37 ± 9.16 years Mean ± SD Height: 180.68 ± 6.12 years Mean ± SD Weight: 90.21 ± 11.59 Mean ± SD BMI ^v^: 27.61 SD 3.09 Healthy Male Police Officers (NZ ^c^)	Fitted stab-resistant body armour and simulated duty gear (7.65 ± 0.73 kg)	IND ^d^: Loaded or unloaded condition, fatigued or unfatigued state DEP ^e^: Balance, grappling, functional mobility task performance, chin up repetitions	Timed stabilometer balance task Simulated vehicle exit/sprint Grappling bag exercise Max rep. Of chin-ups	• Time off balance increased 2.42 s on average when loaded (*p* < 0.001) • Average acceleration time increased 0.28 s on average when loaded (*p* < 0.001) • Average TTC ^h^ for grappling bag exercise increased 1.89 s on average when loaded (*p* < 0.001) • 2.86 Fewer chin-ups were completed when loaded (*p* < 0.001)	78.6
Dempsey, et.al 2014 [36] Body Armour: the effect of load exercise and distraction on landing forces	*n* = 52 Mean ± SD Age: 37 ± 9.16 years Mean ± SD Height: 180.68 ± 6.12 years Mean ± SD Weight: 90.21 ± 11.59 Mean ± SD BMI ^v^: 27.61 SD 3.09 Healthy Male Police Officers (NZ ^c^)	Fitted stab-resistant body armour and simulated duty gear (7.65 ± 0.73 kg)	IND ^d^: Loaded or unloaded condition, fatigued or unfatigued state DEP ^e^: Ground reaction force, jump height	Fatigued and non-fatigued force plate measurements of a max vertical jump, drop landing, predictable and unpredictable jump tasks following drop landing	• Jump height reduced when loaded (*p* < 0.001) • GRF increased when loaded (*p* < 0.001) • Fatigue contributes additional GRF and jump height loss (*p* < 0.001)	85.7
Larsen, et. al. 2012 [34] Body Armour, Performance, and Physiology During Repeated High-Intensity Work Tasks	*n* = 11 Mean ± SD Age: 22 ± 2 years Mean ± SD Height: 185 ± 10 cm Mean ± SD Weight: 77 ± 14 kg Healthy, recreationally active males University Students (US ^p^)	Protective chest plate with integral extremity protection (16.98 ± 1 kg) Military helmet (0.5 kg)	IND ^d^: Armoured or unarmoured condition DEP ^e^: Shooting accuracy, vaulting, crawling, box lifting, body temperature	Timed trial of military task circuit course	• Body Temperature increased in the armoured condition (0.50 ± 0.41 °C) (*p* = 0.02) • RPE ^f^ was 1 ± 0 higher in the armoured condition (*p* < 0.01) • No other test variables reached statistical significance (*p* > 0.05)	75.0
Lenton, et. al. 2015 [35] The effects of military body armour on trunk and hip kinematics during performance of manual handling tasks	*n* = 16 Mean ± SD Age: 22 ± 1 Mean ± SD Height: 180 ± 8 cm Mean ± SD Weight: 74.9 ± 7.5 kg Healthy, active male University students (AU ^b^)	Modular Combat Armour System (8.6 kg) Tiered Body Armour System 1(7.0 kg) Tiered Body Armour System 2(7.3 kg) Tiered Body Armour System 3(6.4 kg)	IND ^d^: Loaded or unloaded condition, body armour configuration worn DEP ^e^: Average flexion angle, trunk rotation ROM ^w^	Ammunition box lift and place Ammunition box Lower and place Sandbag lift and place Each task assessed with a 12 camera motion capture system	• No significant differences between armour configuration (*p* > 0.05) • Any loaded condition significantly decreased trunk rotation ROM ^w^ (*p* < 0.001) • Any loaded condition increased flexion (*p* < 0.001)	60.7
Majchrzycka, et. al. 2013 [28] Ergonomics Assessment of Composite Ballistic Inserts for Bullet- and Fragment-Proof Vests	*n* = 10 Mean ± SD Age: 32.07 ± 2.1 year Mean ± SD Height: 183.0 ± 6.8 cm Mean ± SD Weight: 85.5 ± 9.8 kg Healthy, military males (Poland)	One of three front ballistic plate inserts only within a standard tactical vest Plate WKP ^x^ 112(2.10 kg) Plate WKP ^x^ 14(3.18 kg) Standard Plate (2.40 kg)	IND ^d^: Which of 3 unique ballistic plates was being worn DEP ^e^: Subjective ergonomics, Grandjean scale, attention/perceptiveness, complex reaction time	Battery of questions, assessment of cognitive function, completion of functional tasks	• Although inconclusive, the lightest plate was rated the highest in subjective scoring • No conclusive changes in cognitive function across the tested plates	75
Majumdar, et. al. 1997 [37] Physiological Effects of Wearing Body Armour on Male Soldiers	Studies I, II: *n* = 6 Mean ± SD Age: 29.3 ± 4.08 years Mean ± SD Height: 166 ± 5.9 cm Mean ± SD Weight: 58.5 ± 6.92 kg Healthy male Soldiers (India) Study III: *n* = 16 Mean ± SD Age: 31.5 ± 5.24 years Mean ± SD Height: 167.9 ± 6.15 cm Mean ± SD Weight: 62.8 ± 6.92 Healthy male Soldiers (India)	11.0 kg metallic plate vest with foam backing (Indian Standard Issue)	IND ^d^: Loaded or unloaded condition DEP ^e^: Treadmill test performance, hot-humid exposure step test performance and pulmonary function	2.22 m/s, 10 m flat treadmill walk 20 m resting followed by 40 m light exercise in 34 °C, 60% humidity conditions Measurement of FE ^j^, FVC ^g^ and max voluntary ventilation	• Physiological values of HR ^k^, and VO2 ^l^ all showed significantly greater exertion in the loaded state (*p* < 0.01) • Pulmonary function deteriorated in the loaded condition (*p* < 0.001)	71.4
Phillips, et. al. 2015 [31] The Effects of Military Body Armour on the Lower Back and Knee Kinematics During Toe-Touch and Two-Legged Squat Tasks	Males: *n* = 12 Mean ± SD Age: 26.67 ± 5.47 years Mean ± SD Height: 179 ± 5 cm Mean ± SD Weight: 78.77 ± 9.41 kg Females: *n* = 12 Mean ± SD Age: 24 ± 5.02 Mean ± SD Height: 166 ± 10 cm Mean ± SD Weight: 61.54 ± 7.38 kg Healthy University Students (US ^p^)	10.81 kg armour setup consisting of front and back ceramic plates only in a military vest	IND ^d^: Loaded or unloaded condition, fatigued or non-fatigued state DEP ^e^: Lower back and Knee kinematics	Kinematic motion capture examinations of Toe-touch and 2-legged squat tasks both before and after 45 m treadmill walking at 1.65 m/s	Pre Exercise: • duration of self-paced motion capture tests increased (*p* ≤ 0.02) • Increased time spent in flexion at both joints (*p* ≤ 0.02) Post-Exercise: • Small but significant effects on duration of motion capture tests (*p* = 0.03) • Increased time spent in flexion at both joints (p ≤ 0.04)	78.6
Phillips, et. al. 2016 [32] The Effects of Military Body Armour on the Lower Back and Knee Mechanics During Box Drop and Prone to Standing Tasks	Males: *n* = 12 Mean ± SD Age: 26.67 ± 5.47 years Mean ± SD Height: 179 ± 5 cm Mean ± SD Weight: 78.77 ± 9.41 kg Females: *n* = 12 Mean ± SD Age: 24 ± 5.02 Mean ± SD Height: 166 ± 10 cm Mean ± SD Weight: 61.54 ± 7.38 kg	10.81 kg armour setup consisting of front and back ceramic plates only in a military vest	IND ^d^: Loaded or unloaded condition, fatigued or non-fatigued state DEP ^e^: Lower back and Knee kinematics	Kinematic motion capture examinations of box drop and prone-to-standing tasks both before and after 45 m treadmill walking at 1.65 m/s	Pre-Exercise: • The loaded state increased GRF ^i^, time to completion and time spent in flexion (*p* ≤ 0.02) Post-Exercise: • Thorax posture was at an increased angle for females and a reduced angle for males (*p* = 0.05)	78.6
Ricciardi, et. al. 2007 [28] Effects of Gender and Body Adiposity on Physiological Responses to Physical Work While Wearing Body Armour	Males: *n* = 17 Mean ± SD Age: 31.9 ± 4.1 year Mean ± SD Height: 173.6 ± 4.8 cm Mean ± SD Weight: 78.5 ± 14.9 kg Females: *n* = 17 Mean ± SD Age: 30.4 ± 4.8 years Mean ± SD Height: 163.8 ± 5.1 cm Mean ± SD Weight: 62.1 ± 9.4 kg Healthy, active, military personnel (US ^p^)	Interceptor Outer Tactical Vest (7.8–11 kg)	IND ^d^: Loaded or unloaded condition DEP ^e^: Graded treadmill test performance, blood lactate levels, physical performance battery outcome	Measurement of physiological metrics and recorded perceived exertion of each subject when performing a graded treadmill & military PT ^m^ test	• All subjects showed decreased performance and increased physiological strain when equipped with body armour (*p* ≤ 0.04) • No significant effect of gender on physiological parameters (*p* > 0.05) • Women reported a higher RPE ^f^ than men (*p* = 0.018)	64.3
Ricciardi, et. al. 2008 [23] Metabolic Demands of Body Armour on Physical Performance in Simulated Stress Conditions	Males: *n* = 17 Mean ± SD Age: 31.9 ± 4.1 year Mean ± SD Height: 173.6 ± 4.8 cm Mean ± SD Weight: 78.5 ± 14.9 kg Females: *n* = 17 Mean ± SD Age: 30.4 ± 4.8 years Mean ± SD Height: 163.8 ± 5.1 cm Mean ± SD Weight: 62.1 ± 9.4 kg Healthy, active, military personnel (US ^p^)	Interceptor Outer Tactical Vest (7.8–11 kg)	IND ^d^: Loaded or unloaded condition DEP ^e^: Treadmill test performance, hand grip strength, stair step test performance and pull-ups/hang-time	Subjects participated in 2 test sessions, either with or without body armour and completed the physical performance battery	• Significantly increased physiological strain during treadmill testing (*p* < 0.001) • Stair stepping and pull-ups/hang time were decreased in the loaded state (*p* < .0025), and no change in handgrip strength was noted	75.0
Roberts, et. al. 2013 [39] The Effect of Exercise and Body Armour on Cognitive Function in Healthy Volunteers	Study 1: *n* = 40 Mean ± SD Age: 20.8 ± 1.7 years Healthy male University Students (US ^p^) Study 2: *n* = 20 Mean ± SD Age: 26.6 ± 6.2 years Healthy male University students (US ^p^)	S203 Tactical Vest F6 PASGT ^n^ Helmet	IND ^d^: Brief or extended exercise, loaded or unloaded condition DEP ^e^: Heart rate, oral temp, cognitive function	Study 1: Determine the level of exercise necessary to observe a change in cognitive function Study 2: Physiological variables and cognitive function	• No effect on oral temperature • Loaded condition significantly increased HR ^k^ (*p* < 0.05) • Time, but not loaded condition affected cognitive performance (*p* < 0.05)	64.3
Sell, et. al. 2013 [30] The Addition of Body Armour Diminishes Dynamic Postural Stability in Military Soldiers	*n* = 36 (4 females, 32 males) Mean ± SD Age: 29 ± 6.6 years Mean ± SD Height: 174.49 ± 8.84 cm Mean ± SD Weight: 82.38 ± 13.93 kg Healthy, active Airborne Infantry Soldiers (US ^p^)	Standard US Army ACU ^q^ Uniform Standard issue US Army Body Armour (Mean ± SD weight 12.47 ± 2.56 kg)	IND ^d^: Loaded or unloaded condition DEP ^e^: Dynamic postural stability	Force plate analysis of a single leg jumping task	• The loaded condition resulted in significantly less dynamic postural stability (*p* < 0.001)	71.4
Swain, et. al. 2010 [29] Effects of Training on Physical Performance Wearing Personal Protective Equipment	Vest Group: *n* = 17 (9 females, 8 males) Mean ± SD Age: 22.8 ± 2.5 years Mean ± SD Height: 171 ± 7 cm Mean ± SD Weight: 72.6 ± 12.9 Mean ± SD BMI: 24.7 ± 3.6 Healthy, active, male university students (US ^p^) Control Group: *n* = 20 (9 females 11 males) Mean ± SD Age: 21.9 ± 2.4 years Mean ± SD Height: 174 ± 8 cm Mean ± SD Weight: 74.8 ± 14.2 kg Mean ± SD BMI: 24.6 ± 3.4 Healthy, active female university students (US ^p^)	Small, medium or large ballistic vest with inserts (US ^p^ standard issue) US ^p^ standard issue ballistic helmet Total ensemble mean ± SD mass: 11.7 ± 2.8 kg	IND ^d^: Training with or without the ballistic vest/helmet DEP ^e^: FEV ^j^, VO2max ^s^, RER ^t^, HRmax ^u^, USMC ^r^ PRT ^o^ values	6 week USMC ^r^-styled physical training regimen either with or without armour	• HRmax ^u^ decreased slightly but significantly (*p* = 0.01) in both groups • VO2max ^s^ increased ~2× for the vest group, but this difference did not reach statistical significance (*p* = 0.16) • No changes in FEV ^j^ • Slight but significant (*p* = 0.01) decrease in RER ^t^ in both groups • Both groups significantly improved PRT scores (push-ups, sit-ups, pull-ups, and 3-mile run) following training (*p* < 0.001)	82.1

Legend by order of appearance

^a^ CAS: Critical Appraisal Score

^b^ AU: Australia

^c^ NZ: New Zealand

^d^ IND: Independent

^e^ DEP: Dependent

^f^ RPE: Rating of Perceived Exertion

^g^ FVC: Forced Vital Capacity

^h^ TTC: Time to Completion

^i^ GRF: Ground Reaction Forces

^j^ FEV: Forced Expiratory Volume

^k^ HR: Heart Rate

^l^ VO2: Volume of Oxygen

^m^ PT: Physical Training

^n^ PASGT: Personnel Armour System for Ground Troops

^o^ PRT: Physical Readiness Test

^p^ US: United States of America

^q^ ACU: Army Combat Uniform

^r^ USMC: United States Marine Corps

^s^ VO2max: Maximal Volume of Oxygen

^t^ RER: Respiratory Exchange Ratio

^u^ HRmax: Heart Rate Maximum

^v^ BMI: Body Mass Index

^w^ ROM: Range of Motion

^x^ WKP: A brand of ballistic armour inserts

### Physiological studies

For those studies investigating the physiological effects of wearing body armour, no clear consensus on the independent impacts of wearing body armour emerged from the quantitative data presented in the reports of these studies, as no objective measures were uniformly utilized across all six studies. Nevertheless, the related measures of cardiovascular strain [33], pulmonary strain [37] and heart rate [36, 37] were all reported as elevated beyond control levels in participants wearing body armour suggesting that workload increased when wearing body armour. The study by Swain, et. al. [29] was unique from the others, in that it considered the potential for wearing body armour during training to increase the benefit of training. After 6 weeks of an United States Marine Corps (USMC) style program, the study showed small improvements to the respiratory exchange ratio (RER) (*p* = 0.01), and maximal heart rate (HRmax) (*p* = 0.01) in both the experimental group, which wore body armour during training, and the control group. Likewise, maximal oxygen uptake (VO2max) increased in both groups, however, whilst mean VO2max increased approximately twice as much in the vest group as in the control group, this difference between groups did not reach statistical significance (*p* = 0.16).

### Marksmanship studies

Carbone et. al. [5] found no statistically significant difference in marksmanship scores between participants in a body armour condition and those in a minimally loaded control condition, during various marksmanship trials. This study tested specialist police officers in both tactically-loaded condition (ballistic vest, helmet, and primary (M4) and secondary (Glock) weapons of approximately 22 kgs) and an unloaded condition (dressed in fatigues only). Although no statistically significant differences were found in marksmanship measures (5-bullet impact distance from centre of target, on the vertical and horizontal axis), the authors did report that there was a trend towards horizontal target groupings being superior in the loaded condition, during both the static and mobile shooting (25 m pursuit) tasks. While this result did not reach statistical significance, the authors hypothesize that because the participants train primarily in the loaded state, their accuracy may be better when loaded due to a practice effect and the potential stabilizing effect of the body armour.

### Mobility studies

Of the two studies [3, 14] investigating changes in mobility associated with wearing body armour, one [14] reported a statistically significant difference between a group wearing a tactically loaded specialist police officer body armour configuration (mean ± SD: 22.8 ± 1.8 kg total equipment weight) and an unloaded control group when testing participants on a 10 m sprint, 25 m simulated patrol, and dummy drags [14]. Loads that exceeded 25% of body weight resulted in a significantly greater effect than lower loads. With respect to task completion, the dummy drag was most severely impacted by load (unloaded mean ± SD time to complete = 9.29 ± 0.53 s: loaded = 10.25 ± 0.77 s). In the other study [3], the researchers found that a stab resistant body armour configuration (mean ± SD weight: 7.65 ± 0.73 kg,) when compared to no load, significantly increased time off balance (mean time 8.12 s loaded, 5.7 s unloaded, when using the stabiliometer, *p* < 0.001), time to completion during a simulated vehicle exit/sprint (mean time 1.95 s loaded, 1.67 s unloaded, *p* < 0.001), and time to completion of a mobility battery (mean time 18.16 s loaded, 15.85 s unloaded, *P* < 0.001).

### Kinematic and kinetic studies

Five Studies [3, 30–32, 35] reporting on trunk mechanics found that wearing body armour either compromised trunk posture [32] (by increasing flexion or extension during the test activities) [31, 32, 35], reduced range of motion [35], or reduced stability [3, 30]. These impacts of wearing body armour (total weight ranging from 6.4–12.5 kg) reached statistical significance (*p* ≤ 0.05). Additional observed kinematic impacts of wearing body armour included: increased time to occupational task completion, ranging from 1.89 s extra for grappling tasks (*p* < 0.001) [3] to 0.5 s extra for both box drop and prone to standing tasks (*p* = 0.03) [32]; reduced jump height (*p* < 0.001) [36]; and increased ground reaction forces (GRF) (6–19% over control figures) (*p* ≤ 0.02) [32, 36].

### Cognition

Of the three included studies that considered the impacts of wearing body armour on cognitive function [33, 38, 39], only the study by Roberts, et. al. [39] found a loss in cognitive function when wearing body armour. In that study, the researchers used a verbal fluency task and a controlled order word association task as outcome measures, and observed changes in cognitive strategy. As participants became fatigued, executive function decreased and non-executive function increased (*p* < 0.05). For this reason, the authors attributed cognitive function decline to fatigue over time, and stated that body armour (S203 Tactical vest and PASGT helmet) did not mediate cognitive impairment, even though clearly it may have contributed to fatigue. Majchrzycka, et. al. [38], found no statistically significant differences between body armour and no-armour groups when comparing the effects of a variety of ballistic chest plates only (2.1–3.2 kg) on cognitive performance, measured via the Grandjean scale, tests of attention and perceptiveness and complex reaction time tests for cognition assessment,. Caldwell et. al. [33] also found no statistically significant relationship between cognitive performance (assessed using the Mini-Cog rapid assessment battery) and wearing body armour (7.36 kg, vest and helmet) (*p* > 0.05).

### Subjective outcomes

All studies considering the rating of perceived exertion (RPE) [28, 33, 34, 36] observed statistically significant increases in RPE during activities undertaken by participants when they were wearing body armour (with loads ranging from 7.8 to 17.48 kg) while performing tasks including shooting accuracy, vaulting, crawling, box lifting and graded exercise testing. One study, using a 7.8–11 kg interceptor plate vest, noted that females reported a higher RPE (1 level of perceived exertion higher on average) than males for the same given tasks [28].

### Thermal outcomes

Two studies [33, 34] found that the loaded state (7.36 kg [33] to 19.48 kg [34]) elevated body temperature during activity (by 0.41 to 0.50 °C), beyond control levels in an environment of 21.3 °C (no relative humidity provided) to 36 °C (60% relative humidity) [33]. Body temperature data were obtained either via the auditory canal [33] and from the rectum or an ingested radio-telemetry pill [34]. Another study [39] found that body temperature was not affected by body armour, but used oral temperature, rather than temperature in the gastro-intestinal tract or temperature assessed via the auditory canal. In this study the weight of the body armour, environmental temperature and humidity were not provided.

## Discussion

The aim of this systematic review of the literature was to identify and critically appraise the methodological quality of published studies that have investigated the impacts of body armour on task performance in tactical populations, and to synthesize and report key findings from these studies to inform law enforcement organizations. In total, 16 publications were critically reviewed, achieving a mean ± SD critical appraisal score of 73.2 ± 6.8% (range 60.7 to 85.7%). Seven emerging categories, based on reported outcome measures, emerged to form the basis of the themes reported in the preceding results section and now discussed in the context of findings from the broader tactical research literature. These seven themes, discussed below, are physiological effects, marksmanship effects, mobility effects, kinetic and kinematic effects, cognitive effects, subjective measures, and thermal effects.

### Physiological effects of wearing body armour

The wide variety of loads (ranging from 7.36 kg [33] to 17.48 kg [34]) and test conditions (e.g., hot/humid exercise conditions [33, 37], simulated functional task circuit [34], graded treadmill testing [28], physical fitness testing [23, 28] and running tasks [39]) resulted in heterogeneous results across studies considered in the physiological study category. Additionally, the available studies in this category did not always use comparable outcome measures – for example, studies used related but distinct measures of cardiovascular strain [33], pulmonary strain [37], body temperature and heart rate [36, 37]. With this variability in outcome measures likely contributing to the observed variability in study findings, specific conclusions that can be drawn from this review about physiological effects of wearing body armour are limited. Nevertheless, the included studies, which analyzed HR, pulmonary function, blood lactate levels, VO2max, and RER, did indicate overall that workload increased when wearing body armour, and this is not surprising since body armour adds load. This finding is consistent with findings of military studies, such as the article by Polcyn, et. al. [41], which found increased energy expenditure in female soldiers during a loaded (12–50 kg) 3.2 km land navigation course. Therefore, the physiological data reported in this review, as well as the subjective data (discussed later in this report) and related outcome measures including mobility, balance, time to complete tasks and other occupationally relevant measures reported in the included studies all indicate that wearing body armour results in additional increases in exertion when LEO perform physically demanding tasks, and this finding is consistent with findings in military populations.

### Marksmanship effects of wearing body armour

Only one study in the marksmanship category assessed the effects of body armour on marksmanship of tactical populations [5]. In this study, using an M4 rifle and Glock handgun, no significant effect of wearing the body armour was found. The authors cited low numbers of participants (*n* = 6) and limited marksmanship data as major contributors to the inconclusive result. Similar challenges were reported by Orr, et. al. [42], who also considered tactical police officers in their research. A further study investigated participants drawn from a military population performing exercise (repetitive 20.5 kg/1.55 m box lift) while wearing a combat load (29.9 kg) [43] and found that while the load did not decrease rifle accuracy, an increase in the time to engage targets occurred. This increase in time to engage targets could impact fighting effectiveness and survivability in a combat environment, regardless of occupation. In other military load carriage studies, such as two investigations by Knapik et. al., [20] and Hanlon [44], a decrease in M16 rifle shooting performance was found after a 20 km road march under loads up to 61 kg, and after a 2 mile run for time. These findings, when taken together, suggest that the independent effect of load carriage on marksmanship is likely to be negative but is also still not well understood, especially when different weapon systems are used (e.g. rifle versus hand gun), warranting further research in this area.

### Mobility effects of wearing body armour

This review found that balance is decreased and time to complete functional tasks is increased as a result of wearing body armour. Additionally, some occupationally relevant tasks may be significantly impaired by body armour. These findings are further supported by the results of previous military research [45] showing increased road march time on completion of a functional task (US Army obstacle course) by subjects wearing a full fighting load (14–27kgs). Additional military-focused research showed significant effects of body armour on road march time and the incidence of load-incited blisters [44]. Moreover, a study investigating body armour and full fighting loads also reported biomechanical disadvantage (decreased trunk range of motion) resulted from increasing the external load. Body armour may contribute to development of musculoskeletal injury and chronic low back pain, both of which are reported as being substantial factors in lost time on duty in tactical settings [10, 46].

### Kinematic and kinetic effects of wearing body armour

The type of body armour selected for use is characterised by three primary factors: level of protection, actuarial concerns, and degree of functional impairment exerted by the system [47, 48]. Since neither level of protection nor cost are enhanced by systems with potential to improve mobility [49], effective body armour has been repeatedly shown to be ergonomically detrimental, specifically with respect to trunk posture [3, 30–32, 35]. GRF [3, 32, 36] universally deteriorated when participants in the included studies were wearing body armour. That is to say, that both fatigue and being in a loaded condition elevated GRF, with the combination of the two resulting in the most impact. With this in mind, a survey of 863 US Soldiers in Iraq [50] found a significant positive correlation between the duration for which Soldiers wore body armour each day and rates of musculoskeletal complaints, such that those who wore the body armour for four hours or more per day were at significantly greater risk. The musculoskeletal complaints ranged from neck and back to upper extremity musculoskeletal pain. For this reason, the weight and ergonomics of body armour systems should be closely evaluated when making equipment decisions.

### Cognitive effects of wearing body armour

Heterogeneity limits cross-study comparisons of cognitive effects of wearing body armour in the broad tactical literature and across the studies included in this review. The studies included in this review used a variety of body armour types and configurations and measures of cognitive performance (e.g., unique word association tests, a variety of measures of fatigue levels, and wide-ranging equipment and loads). Nevertheless, one key finding from the included cognition studies is the finding that time and fatigue induced deteriorations in cognitive performance when personnel were carrying loads, including body armour [39]. If an armoured state can bring about fatigue more rapidly than a control state, as has been suggested by several pertinent studies included in this review [3, 23, 28, 36], then relationships between the armoured state, fatigue and cognition can be established. Other research, such as one study of ROTC cadets carrying an external load in the form of all-purpose, lightweight, individual, carrying equipment (ALICE) backpacks [51], has reported significant deterioration in executive, higher-level, mental processing when carrying load (examining tests of situational awareness wearing 30% of body weight). This finding, in conjunction with the aforementioned findings of this review, could be significant for both LEO and military populations, where critical thinking and decision making skills are vital, often for extended periods of time and under significant levels of stress.

### Perceived impacts of body armour

Increases in RPE reported by participants when wearing body armour, when compared to their unloaded peers or unloaded time periods, revealed that even in the absence of quantitative measurement of physiological effects, individuals equipped with armour perceived more required effort in completing a task when wearing body armour, regardless of whether or not the subjects recruited were from a tactical background. These results were consistent with other research within the general field of load carriage where increased loading increase subjective ratings of effort [52]. In contrast, one review by Larsen, et. al. [53] found no consensus across reviewed study reports regarding exertion effects of wearing body armour, but this is likely because research considering non-tactical (Emergency Medical Services, firefighting) populations was included in the search strategy.

### Thermal effects of wearing body armour

While increases observed in the included studies in core temperature due to wearing body armour were mild (0.5 °C), it should be emphasized that these outcomes were assessed *independently* of the physical activities within the study protocol. Specifically, time exposed to the hot-humid test condition and loaded state proved to be the variables most strongly associated with temperature deviation from the control values [33]. Therefore, although core temperatures associated with heat illness were not found in data obtained through this review, personnel operating in high heat and high humidity environments should receive special consideration with respect to exertional heat illness and limits on time on duty when equipped with body armour.

### Limitations

One notable limitation of this review was the generally small number of participants in each study (ranging from 6 to 52 subjects, mean = 22.16, median = 17). Furthermore, when each sub field was considered in isolation, the relative participant size decreased further (e.g. thermal effects participant sizes of 9 [33] and 11 [34]). On this basis, the results should be considered with caution until larger studies (or a greater summation of smaller studies) can provide further supporting evidence.

## Conclusions

Based on research quality and agreement across studies, this review determined that while effects of wearing body armour on marksmanship and various physiological parameters are still uncertain and deserving of further research, body armour does have significant biomechanical and physical performance impacts on the wearer, including: a) increased workload as assessed by a range of different physiological measures, b) decreased work capacity (measured as fitness test score deterioration), b) increased time to complete functional tasks, c) decreased balance and stability, d) increased GRF, e) increased RPE, and f) mild elevation of core temperatures. These occupationally-relevant performance decrements may also lead to decreased cognitive capability and increased injury risk. For this reason, body armour should be carefully selected, with consideration of the levels of physical conditioning of the wearers and the degree to which the armour system can be ergonomically optimized for the individual wearer.

## Abbreviations

ALICE: All-purpose, lightweight, individual, carrying equipment
CAS: Critical appraisal score
CBRNE: Chemical, biological, radiological, nuclear, and high-yield explosive
GRF: Ground reaction forces
HRmax: Heart rate maximum
LEO: Law enforcement officer
PPE: Personal protective equipment
RER: Respiratory exchange ratio
ROTC: Reserve officer training corps
RPE: Rate/Rating of perceived exertion
